# Indirect Volume Estimation for Acute Ischemic Stroke from Diffusion Weighted Image Using Slice Image Segmentation

**DOI:** 10.3390/jpm12040521

**Published:** 2022-03-24

**Authors:** Seung-Ah Lee, Jae-Won Jang, Sang-Won Park, Pum-Jun Kim, Na-Young Yeo, Chulho Kim, Yoon Kim, Hyun-Soo Choi, Seongheon Kim

**Affiliations:** 1 Department of Computer and Communications Engineering, Kangwon National University, Chuncheon 24253, Korea; a985060@kangwon.ac.kr; 2 Department of Medical Bigdata Convergence, Kangwon National University, Chuncheon 24253, Korea; light26@kangwon.ac.kr (J.-W.J.); chicwon229@kangwon.ac.kr (S.-W.P.); 3 School of Medicine, Kangwon National University, Chuncheon 24253, Korea; 4 Department of Neurology, Kangwon National University Hospital, Chuncheon 24253, Korea; 5 Institute of New Frontier Research Team, Hallym University College of Medicine, Chuncheon 24252, Korea; pumjun4093@gmail.com; 6 Chuncheon Artificial Intelligence Center, Chuncheon Sacred Heart Hospital, Chuncheon 24253, Korea; nayung91@gmail.com; 7 Department of Neurology, Chuncheon Sacred Heart Hospital, Chuncheon 24253, Korea; gumdol52@naver.com; 8 Department of Computer Science and Engineering, Kangwon National University, Chuncheon 24253, Korea; yooni@kangwon.ac.kr; 9 ZIOVISION, Chuncheon 24341, Korea

**Keywords:** acute ischemic stroke, computer-aided diagnosis, segmentation, deep-learning

## Abstract

The accurate estimation of acute ischemic stroke (AIS) using diffusion-weighted imaging (DWI) is crucial for assessing patients and guiding treatment options. This study aimed to propose a method that estimates AIS volume in DWI objectively, quickly, and accurately. We used a dataset of DWI with AIS, including 2159 participants (1179 for internal validation and 980 for external validation) with various types of AIS. We constructed algorithms using 3D segmentation (direct estimation) and 2D segmentation (indirect estimation) and compared their performances with those annotated by neurologists. The proposed pretrained indirect model demonstrated higher segmentation performance than the direct model, with a sensitivity, specificity, F1-score, and Jaccard index of 75.0%, 77.9%, 76.0, and 62.1%, respectively, for internal validation, and 72.8%, 84.3%, 77.2, and 63.8%, respectively, for external validation. Volume estimation was more reliable for the indirect model, with 93.3% volume similarity (VS), 0.797 mean absolute error (MAE) for internal validation, VS of 89.2% and a MAE of 2.5% for external validation. These results suggest that the indirect model using 2D segmentation developed in this study can provide an accurate estimation of volume from DWI of AIS and may serve as a supporting tool to help physicians make crucial clinical decisions.

## 1. Introduction

Acute ischemic stroke (AIS) is a major cause of disability, and an accurate and rapid decision for treatment strategies is critical to improving patient outcomes [1,2]. Time is regarded as the most important factor in acute stroke triage, and reperfusion by thrombectomy with or without intravenous tissue plasminogen is the only way to successfully reverse ischemic changes [3]. Recent clinical trials, such as Extending the Time for Thrombolysis in Emergency Neurological Deficits (EXTEND) [4], Diffusion and Perfusion Imaging Evaluations for Understanding Stroke Evolution Study (DEFUSE) [5], and Diffusion-weighted imaging (DWI) or Computerized Tomography Perfusion Assessment with Clinical Mismatch in the Triage of Wake-Up and Late Presenting Strokes Undergoing Neurointervention with Trevo (DAWN) [6], have demonstrated the value of evaluating the infarct volume and viable brain tissue on brain imaging. DWI is a commonly used magnetic resonance imaging (MRI) sequence with high sensitivity and a short acquisition time [7]. The initial signal change in DWI of AIS can indicate the infarct core that correlates with the final infarct volume; accurate segmentation of acute lesions on DWI is essential for guiding treatment options and evaluating patients [7,8].

Various high-performance automatic segmentation methods have been developed for DWI of AIS [9,10,11], but their high dependence on handcrafted features limits their modeling capabilities. Additionally, AIS remains challenging due to various subtypes, many artifacts, multifocal distribution, and ill-defined stroke boundaries [12]. Detecting DWI in AIS is crucial for diagnosis and treatment planning, but this task is laborious, time-consuming, and can involve physicians’ subjectivity.

This study aimed to propose a method that estimates AIS volume in DWI objectively, quickly, and accurately. The proposed method performs segmentation on DWI and estimates the volume of the segmented AIS.

## 2. Materials and Methods

### 2.1. Study Population

Consecutive patients who underwent DWI for AIS were retrospectively recruited from two hospitals (Hallym University Chuncheon Sacred Heart Hospital (HUCSHH) and Kangwon National University Hospital (KNUH)). The AIS cases in each hospital were as follows: from January 2011 to December 2019 for HUCSHH, and from July 2014 to October 2019 for KNUH. Eventually, 2159 DWI images of AIS (for HUCSHH, 694 men and 485 women with a mean ± standard deviation age of 69.8 ± 12.7, and for KNUH, 555 men and 425 women with a mean ± standard deviation age of 72.4 ± 12.4) were included. Additionally, 121 DWI images of control subjects (for HUCSHH, 50 men and 52 women with a mean ± standard deviation age of 59.2 ± 16.4, and for KNUH, 10 men and 11 women with a mean ± standard deviation age of 44.0 ± 17.1) were included in the evaluation of the developed segmentation algorithm. These DWIs were of healthy participants or those whose *b*-value of 1000 s/mm^2^ showed no lesions, whereas a *b*-value of 0 s/mm^2^ might show some old lesions. This retrospective study was approved by the Institutional Review Boards of HUCSHH and KNUH, which waived the requirement for informed consent (approval no. HUCSHH 2021-06-013 and KNUH-A-2021-021-001).

### 2.2. Imaging Acquisition

MRI was performed with various machines, including a 1.5T (Magnetom Avanto, Siemens Healthineers, Erlangen, Germany) and a 3T scanner (Ingenia CX, Philips Healthcare; Achieva, Philips Healthcare, Best, The Netherlands). The parameters for the DWI sequences were as follows: repetition time, 3000 ms–8000 ms; echo time, 56 ms–103 ms; flip angle, 90°; matrix, 256 × 256–512 × 512; field of view, 220 × 220–256 × 256 mm; the number of excitations, 1–5; the number of slices, 20–50; slice thickness, 3 mm; *b*-value, 1000 s/mm^2^.

### 2.3. Annotation

**Figure 1:**
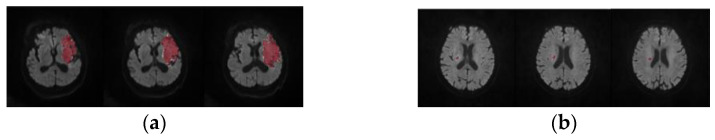
Example of (**a**) large-sized AIS on DWI and (**b**) small-sized AIS on DWI.

All lesions on DWI were manually segmented by one neurologist (C.K) in HUCSHH and by two neurologists (S.H.K and J.-W.J) in KNUH using ITK-Snap, an open-source software application used to segment structures in medical images (http://www.itksnap.org. accessed on 30 December 2020). All images with masks were saved in Digital Imaging and Communications in Medicine (DICOM) format. Figure 1 shows examples of segmented images for large-sized AIS and small-sized AIS.

### 2.4. Method

**Figure 2:**
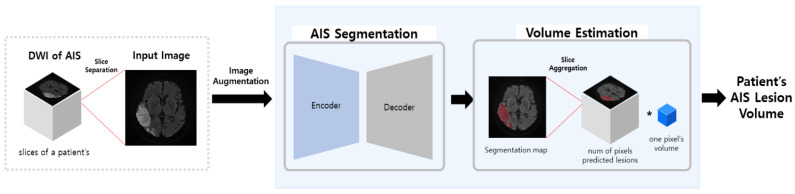
Flowchart of our proposed method.

The method we proposed to execute the volume estimation for AIS is shown in Figure 2. It follows the process of image augmentation, a training segmentation model, and volume estimation using pixels/voxels.

#### 2.4.1. Augmentation

**Figure 3:**
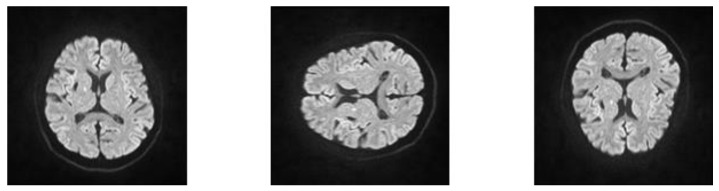
Example of data augmentation.

Training segmentation models require a large amount of data. However, it is difficult to train segmentation models sufficiently because our data are lacking. To address the lack of data, we applied data augmentation. We executed a random horizontal/vertical flip and random 90° rotation [13,14]. Figure 3 shows the results of augmentation. The data were resized to 256 × 256 to shorten the training time and reduce the computational cost.

#### 2.4.2. Volume Estimation

- Direct Volume Estimation (Using 3D Segmentation)

Direct volume estimation was executed by a 3D U-Net [15], an architecture based on convolutional neural networks (CNNs) to process volumes. The 3D U-Net has a U-shaped structure and employs a symmetric encoder and decoder network with skipped connections. The encoder path determines the context by downsampling the spatial dimensions. The encoder consists of three layers: two 3D convolutions, ReLU (rectified linear unit) and batch normalization, and a 2 × 2 max-pooling layer. The decoder path performs upsampling for the encoder output image to increase the spatial dimensions. The decoder consists of three layers, including two 3D convolutions, ReLU and batch normalization, and 2 × 2 up-convolution. Symmetric blocks are used with skip connections from the corresponding encoding blocks to replenish the lost context. The skip connection improves the data recovery performance by adding a layer input to the output to compensate for the information lost during the convolution process and helps to perform semantic segmentation well. The training of 3D segmentation models requires a high computational time and memory. Therefore, we performed transfer learning by using pretrained models with an auto implant and tumor, respectively, and performed patch-based 3D segmentation to alleviate this problem. In the data, the size of the AIS was relatively small compared to the size of the brain and was sparsely located. Extracting small patches from these data causes a problem in that AIS cannot be contained in the patch, resulting in class imbalance. We proposed indirect volume estimation using 2D segmentation, described in the subsequent section, to mitigate the imbalance problem.

- Indirect Volume Estimation (Using 2D Segmentation)

Indirect volume estimation measures the volume of a slice image after changing the volumetric image to a slice image. By dividing the volumetric image into each cross-sectional image and using it as an input for the model, the amount of data increases, and the overall brain cross-sectional information can be viewed, mitigating hierarchical imbalances and data shortages. Indirect volume estimation is executed by 2D U-Net, which has the same model architecture as 3D U-Net but uses 2D convolutions instead of 3D convolutions. In the previous method [16], one channel was used as the input. However, Buda et al. [17] used two adjacent slices as a three-channel input to provide additional information, such as context information and volumetric information. Similarly, our model also used three input channels. Furthermore, we performed transfer learning by using a pretrained model with glioma to improve the model’s performance and save time for the model to train AIS.

**Figure 4:**
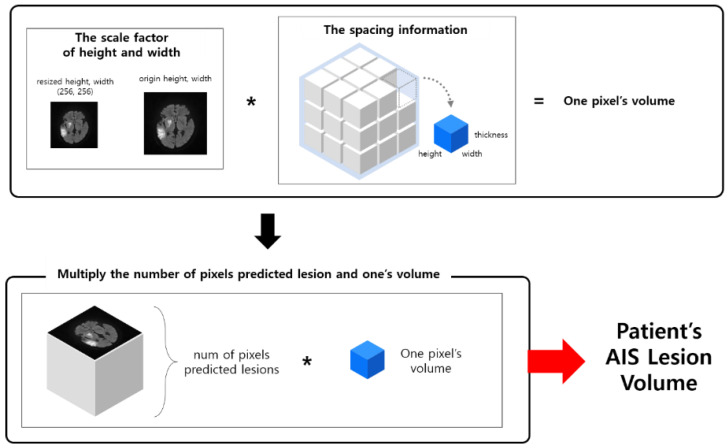
Flowchart of volume estimation.

To estimate the volume indirectly, we used the pixels of the mask predicted as AIS. The detailed algorithm for estimating the volume using the predicted mask is shown in Figure 4.

To obtain the actual volume, the scale factors, $sf_{w},\;sf_{h}$, for the width and height of the original image, respectively, are determined from the resized mask size. The spacing information, si, was obtained by referring to space between slices and slice thickness in the DICOM header file. The volume of a pixel, V, is obtained by multiplying each scale factor and the spacing information as follows:

(1) $$V=sf_{w}*\;sf_{h}*si.$$

By letting H, W, N be the height, width and number of slices, respectively, we obtained the total number of pixels, P, by cumulating all the predicted binary pixels, p, in all slices, as:

(2) $$P=\sum_{n=1}^{N}\sum_{h=0}^{H}\sum_{w=0}^{W}p_{h,w}^{n}.$$

The patient’s AIS volume was obtained by multiplying the volume, V, of one pixel by the total number of pixels, P.

#### 2.4.3. Loss Function

The Dice coefficient [18] is used to compare the areas between the prediction mask and label and is adopted as a loss function, known as Dice loss:

(3) $$L_{dice}=1-\frac{2*\sum y_{true}*y_{pred}}{\sum y_{true}^{2}+\sum y_{pred}^{2}},$$

We trained a model with Dice loss, and the model trained knowledge, such as DWI of AIS. Where $y_{true}\;$ is the label for the pixels/voxels value and $y_{pred}\;$ is the predicted probability for the pixel/voxel value.

#### 2.4.4. Evaluation Metrics

To evaluate the segmentation models, we adopted the sensitivity, specificity, F1-score, and Jaccard index as follows:

(4) $$\mathrm{Sensitivity}=\frac{TP}{TP+FN},$$

(5) $$\mathrm{Specificity}=\frac{TP}{TP+FP},$$

(6) $$F1\;\mathrm{score}=2*\;\frac{\mathrm{Recall}*\mathrm{Precision}}{\mathrm{Recall}+\mathrm{Precision}},$$

(7) $$\mathrm{Jaccard}\;\mathrm{Index}=\frac{\left|\mathrm{prediction}\;\cap \;\mathrm{label}\right|}{\left|\mathrm{prediction}\;\cup \;\mathrm{label}\right|}.$$

Because our study focused on the importance of accurate AIS segmentation, we mainly set metrics for true-positive (*TP*). In Equations (4)–(7), *TP* is the number of outcome pixels correctly predicted as AIS. False-positive (*FP*) is the number of outcome pixels mispredicted as AIS, and false-negative (*FN*) is the number of outcome pixels mispredicted as non-AIS.

To evaluate the volumes, we adopted the volume similarity (*VS*) [19] and mean absolute error (*MAE*):

(8) $$VS=1-\frac{\left|\left|\mathrm{measurement}\right|-\left|\mathrm{groundtruth}\right|\right|}{\left|\mathrm{measurement}\right|+\left|\mathrm{groundtruth}\right|}=1-\frac{\left|FN-FP\right|}{2TP+FP+FN},\;$$

(9) $$MAE=\frac{1}{n}\sum_{i=1}^{n}\left|\mathrm{measurement}-\mathrm{groundtruth}\right|.$$

*VS* estimated the volume of the predicted AIS to indicate its similarity with the labeled AIS. *MAE* is the average of the absolute values of the errors that are the difference between the actual and predicted values, i.e., it estimates the error between the predicted AIS and labeled AIS. In Equations (8) and (9), the measurement is the region of the predicted AIS (*TP*), and the ground truth is the region of the labeled AIS.

## 3. Results

### 3.1. Implementation Details

All methods were implemented using the Pytorch framework 1.10 in Python 3.8.10 on Ubuntu, using four NVIDIA RTX 3090s, and have been included as core algorithms in AI-based medical solutions (ZioMed; ZIOVISION, Chuncheon, Korea). Internal validation was performed using data acquired from HUCSHH. We randomly split the dataset into training, validation, and testing sets at a ratio of 8:1:1. Data acquired from KNUH were used for external validation. Loading the image and spacing information of the dataset is time-consuming. Considering that it takes a long time to load the DICOM file, the image and spacing information extracted from the DICOM file were saved in HDF5.

### 3.2. Segmentation Performance

We conducted an experiment to evaluate the methods for the segmentation performance of DWI in AIS. Table 1 and Table 2 show the performance results of the model that segment the DWI of AIS for internal and external validation. Regarding the internal validation data of patients with AIS, the F1-score of the indirect segmentation with a pretrained model for brain glioma [17] was 76.02%. This was higher than those of the scratch-based indirect volume estimation model (73.09%), the scratch-based direct volume estimation model (54.76%), and direct volume estimation with a pretrained model for auto implants and tumors (55.71%, 52.48%) [20]. Regarding the external validation data of patients with AIS, the F1-score of indirect segmentation with a pretrained model for brain glioma [17] was 77.23%, which was higher than that of the scratch-based indirect segmentation model (73.93%), the scratch-based direct segmentation model (63.94%), and direct segmentation with a pretrained model for auto implants and tumors (56.73%, 60.33%) [20]. Similarly, the Jaccard indices of internal and external validation with pretrained indirect models were 62.12% and 63.82%, respectively, and higher than those of the other models.

### 3.3. Volume Estimation

Through VS and MAE, we confirmed whether the result of estimating the AIS volume with the proposed model is reliable. Table 3 shows the results of estimating the volume of the AIS segmented for internal and external validations. The results of direct volume estimation with a pretrained model for a brain tumor, VS, and MAE in internal validation were 67.68% and 1.159 cc, and in external validation, were 62.59% and 5.706 cc, respectively. Moreso, for indirect volume estimation with a pretrained model for brain glioma, VS and MAE in internal validation were 93.25% and 0.797 cc, and in external validation, were 89.17% and 2.468 cc, respectively.

In addition, to check for FP errors, our best model (indirect pretrained) was validated in the normal group that had no AIS volume at all. For internal and external validation, the MAE values were 0.028 cc and 0.009 cc, respectively.

## 4. Discussion

**Figure 5:**
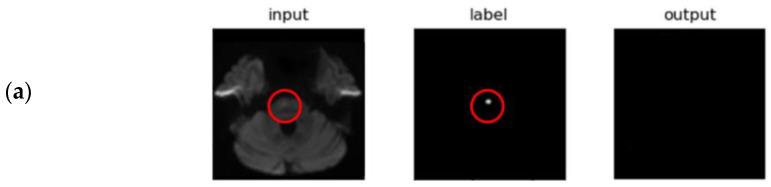

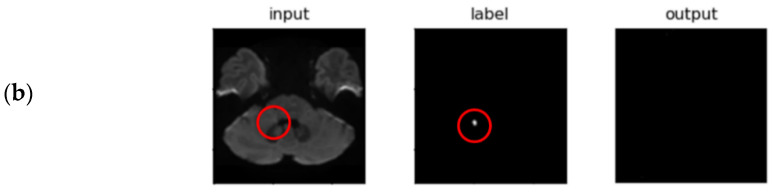
Example images for small-sized AIS: (**a**) Internal validation and (**b**) External validation.

**Figure 6:**
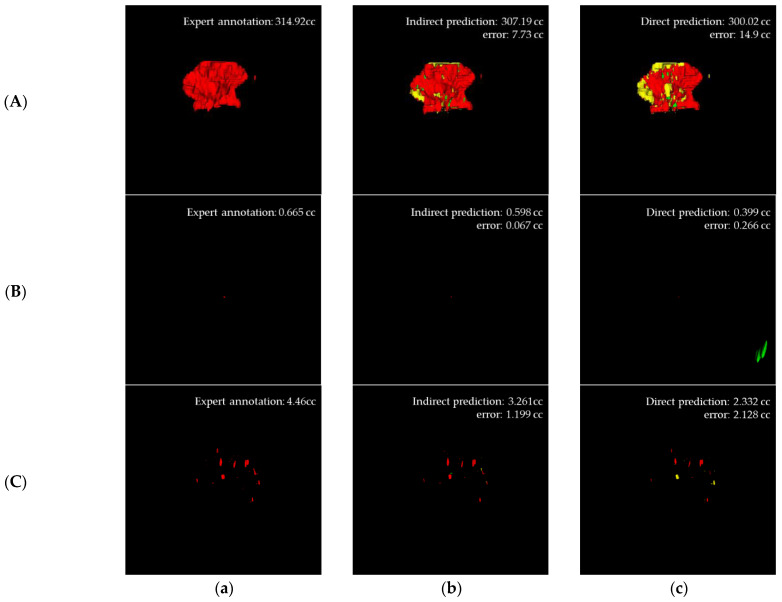
Visual results for (**A**) large AIS, (**B**) small AIS and (**C**) sparsely located small AIS (*TP* is red, *FP* is green and *FN* is yellow): (**a**) Expert annotation, (**b**) Predicted indirect volume, and (**c**) Predicted direct volume.

We proposed a novel method to estimate AIS volume and help doctors make quick decisions accurately. The proposed method is an indirect volume estimation method that estimates AIS volume by converting a volumetric image into a slice image; the volume is estimated using pixels predicted as AIS in each slice. This approach is an effective and attractive method with the advantage of low computational cost. We conducted internal and external validations to confirm this remarkable performance. Table 1 and Table 2 show the performance of AIS segmentation. The internal validation F1-scores of the direct and indirect segmentations were 55.71% and 76.02%, respectively, while the external validation F1-scores of the direct segmentation model and indirect segmentation model were 63.94% and 77.23%, respectively. Although direct segmentation on volumetric data was expected to be high, surprisingly, this study recorded better performance for indirect segmentation by converting volumetric data into slice data (Figure 6A). The reason for this result could be explained by the following:

First, the image information was different for each image. Heterogeneity exists because of the devices used in each hospital and the option settings in the devices (e.g., 1.5T, 3T), noise, etc., which can make it difficult to train models for indirect volume estimation and direct volume estimation. Additionally, the volume shape of patients depends on the number of slices, pathological lesions and slice width [21]. To solve the problem of inconsistent information and image shape, the input image is made consistent by resizing, padding, cropping on the image, or matching the shape. However, in this process, small AIS with small axis pixels, such as Figure 5, Figure 6B, and Figure 6C are deformed, making it difficult to segment AIS.

As mentioned in 2.4 Method, patch-based 3D segmentation was performed on direct volume estimation. This patch-based 3D segmentation trains a model by transforming an input 3D image into 3D subsamples of arbitrary size. However, patch-based 3D segmentation may not be able to extract global features considering the actual image volume, so the size of the AIS to be segmented like ours is relatively small compared to the size of the brain and may show poor results when located infrequently. In addition, class imbalance may occur if an inappropriate patch size is used. Due to these limitations of path-based 3D segmentation, it can be inferred that the training result of direct volume estimation is worse than indirect volume estimation.

In the case of direct segmentation, there was no significant performance difference in the F1-score between the estimated scratch model and the pretrained model. In the case of indirect volume estimation, the pretrained model had a higher F1-score than the scratch model. The reasons for these results are as follows:

First, what the pretrained model predicts is different. In direct volume estimation, the pretrained model for auto implants predicts the shape and size of an implant through an image of a defective skull, unlike ours, which segments lesions through brain images. Moreso, the number of modalities and classes used in the pretrained model is different from ours. In direct volume estimation, brain tumor segmentation of the pretrained model is for segmenting brain tumors composed of multiple labels, like activators and edema, by inputting multimode MRI, such as FLAIR (fluid-attenuated inversion recovery), T1, and T2. This is different from our study, which involves segmenting AIS lesions by inputting only DWI. Therefore, the result of the F1-score seems poor. However, in indirect volume estimation, the pretrained model that segments gliomas by inputting only FLAIR is similar to our study, which segments only one lesion. Hence, the pretrained model outperformed the scratch model in terms of transfer learning effects.

Table 3 shows the results of estimating the actual volume with our proposed model pretrained with glioma, which showed the best performance. These results indicate that the indirect volume estimation shows higher accuracy than direct volume estimation and is somewhat stable and significant. As a result, considering only the F1-score of the AIS segmentation performance, external validation seems to have shown better performance than internal validation; however, internal validation showed better performance in volume estimation. The reason is presumed to be as follows. According to Table 1 and Table 2, focusing on sensitivity that accurately identifies AIS pixels, and specificity that accurately identifies non-AIS pixels, external validation has high sensitivity but low specificity. Internal validation does not show a significant difference between sensitivity and specificity. In other words, in the external validation, pixels other than AIS were well identified, but the actual AIS pixel was not well identified. Hence, the error appeared larger when the volume was measured. These results appear to have been caused by the abovementioned problems for the following reasons. When performing AIS segmentation, internal validation was performed by one neurologist, but two neurologists performed external validation; therefore, the segments were expected to be inconsistent due to their respective subjectivity. To solve the problem of subjectivity in this situation, the proposed model is required.

In AIS, estimating infarction volume is crucial for properly managing patients regarding clinical decisions and predicting outcomes [22,23]. Delayed treatment is well known to be associated with worse cerebral injury, and it is said that “time is brain” [1,24]. According to recent clinical trials, endovascular treatment has become the standard treatment for patients with AIS owing to large arterial occlusion [4,5,6]. In the DAWN trial, which extended the thrombectomy window from 6 h to 24 h, patient selection was based on the mismatch between clinical deficit and the specific infarct volume [6]. The DEFUSE 3 trial, which extended the thrombectomy window to 6 h to 16 h, also adopted an initial infarct volume of <70 mL and a ratio of the volume of ischemic tissue on perfusion imaging to infarct volume of ≥1.8 for endovascular therapy candidates [5]. In the EXTEND trial, which extended the thrombolysis window up to 9 h after the onset of stroke, perfusion lesion-ischemic core mismatch was defined as 1.2, an absolute difference in the volume >10 mL, and an ischemic core volume <70 mL [4]. Therefore, fast decision-making for the selection of patients for endovascular therapy might be supported by the precise and rapid estimation of infarct volume to produce maximal clinical outcomes [25]. Our model, which uses indirect volume estimation, providing a precise estimation of the volume of AIS lesions, could serve as a marker for selecting patients in endovascular treatment and predicting prognosis. Additionally, one of the most important future goals might be the implementation of a model in clinical practice, such as the critical pathway of AIS. In line with this, our model has a limitation in that it is based on DWI of MRI that is available from relatively larger centers. Thus, this study could serve as the starting point for reducing the door to needle time based on future studies to validate with other imaging modalities, such as angiography or computed tomography.

In conclusion, indirect volume estimation showed better results than direct volume estimation. However, indirect volume estimation may encounter difficulty in segmenting sparse and small-sized AIS because the small-sized AIS does not provide many axial slices useful for learning segmentation modes [21]. In future studies, we will focus on improving the performance of small AISs by direct volume estimation. In addition, we will research and develop a system that can predict the degree of the patient’s modified Rankin Score, which can make complex evaluations along with image information by reflecting the patient’s clinical information.

## Figures and Tables

**Table 1:** Segmentation performance for internal validation.

Model		Sensitivity (%)	Specificity (%)	F1-Score (%)	Jaccard Index (%)
Direct	Scratch	75.27	48.02	54.76	42.02
	Pretrained (Auto Implant)	71.00	49.31	55.71	43.04
	Pretrained (Tumor)	63.72	52.27	52.48	39.88
Indirect	Scratch	71.11	75.87	73.09	58.49
	Pretrained (Glioma)	75.0	77.87	76.02	62.12

**Table 2:** Segmentation performance for external validation.

Model		Sensitivity (%)	Specificity (%)	F1-Score (%)	Jaccard Index (%)
Direct	Scratch	70.99	63.66	63.94	51.48
	Pretrained (Auto Implant)	63.22	58.65	56.73	44.93
	Pretrained (Tumor)	67.05	62.26	60.33	48.33
Indirect	Scratch	69.35	83.75	73.93	60.28
	Pretrained (Glioma)	72.81	84.33	77.23	63.82

**Table 3:** AIS Volume estimation results for internal validation and external validation.

Model		VS (%)	*MAE* (cc)
External validation	Direct	62.59	5.706
	Indirect	89.17	2.468
Internal validation	Direct	67.68	1.159
	Indirect	93.25	0.797

## Data Availability

The data presented in this study are available upon request from the corresponding author.
